# Review of *Lophomyra* Schaus, 1911 (Lepidoptera, Noctuidae): a new combination and re-descriptions of species newly associated with ferns (Polypodiaceae)

**DOI:** 10.3897/zookeys.788.21235

**Published:** 2018-10-08

**Authors:** Paul Z. Goldstein, Daniel H. Janzen, Benjamin Proshek, Tanya Dapkey, Winnie Hallwachs

**Affiliations:** 1 Systematic Entomology Laboratory, USDA, National Museum of Natural History, E-502, P.O. Box 37012, MRC 168, Washington, DC 20013-7012 National Museum of Natural History Washington United States of America; 2 Department of Biology, University of Pennsylvania, Philadelphia, PA 19104 University of Pennsylvania Philadelphia United States of America

**Keywords:** Costa Rica, DNA barcode, *
Lophomyra
*, pteridivore

## Abstract

*Lophomyra* Schaus, 1911 (Noctuidae) is the smaller of two noctuid genera originally described by Schaus that include species recently associated with ferns (Pteridophyta), in this case Polypodiaceae, as larval food plants. Following an examination of type material and reared specimens accompanied by DNA barcode data, *Lophomyra* is revised to include *L.tacita* Schaus, 1911, *L.santista* (Jones, 1914), and *L.commixta* (Schaus, 1914), **comb. n.**, the last of which is transferred from *Chytonidia* Schaus, 1914 (= *Leucosigma* Druce, 1908). *Lophomyra* is characterized based on adult and larval morphology, especially that of the male genitalia. Structures associated with the valvae are discussed with reference to dissected and *in situ* images. Larvae of *L.commixta* and *L.tacita* are described from images, and the recorded food plants of both species are discussed in the context of known New World noctuid pteridivores.

## Introduction

*Lophomyra* Schaus, 1911 is one of two noctuine genera (Lepidoptera: Noctuidae: Noctuinae) described by Schaus with larvae recently discovered feeding on fern foliage (Pteridophyta) at Área de Conservación Guanacaste (ACG), northwestern Costa Rica. Because of their age and toxicity, ferns and their associated herbivore faunas have been of interest to plant-insect biologists for decades. In part because some fern-feeding insect groups have only recently become known as such and are in need of systematic treatment, the number of origins of pteridivory has likely been underestimated and the diet breadths of pteridivorous insects oversimplified. Sampling of Lepidoptera larvae during the last four decades in ACG (Janzen and Hallwachs 2016) have identified numerous pteridivorous caterpillars, most of them apparent fern specialists and several of which are species new to science. This paper represents an incipient effort to characterize the prevalence of lepidopteran fern-feeding behaviors, as well as to serve the larger aims of Neotropical moth systematics. Our primary purpose is to better diagnose *Lophomyra*, effect the transfer of *commixta* from *Chytonidia* Schaus, 1914 (=*Leucosigma* Druce, 1908), and figure the larvae of two species while characterizing their host plants at ACG.

## Materials and methods

Pinned specimens were examined with an incandescent light source. Genitalic preparations follow Clarke (1941) in part and Lafontaine (2004), but staining with chlorazol black and mounted in euparal, with the vesicae everted in water prior to fixation. Dissections followed either an overnight room-temperature soak in supersaturated sodium hydroxide or a 15 minute heated soak, and were examined under dissecting microscopes prior to slide-mounting. Photographs were made using the Microptics and Visionary Digital imaging systems and images manipulated with Adobe Photoshop (Adobe Systems, Mountain View, CA). Higher-resolution images are available ffrom the corresponding author. Measurements were made with the aid of an ocular micrometer. Forewing length was measured from the center of the axillary area to the apex of the forewing. Terminology follows Forbes (1954) and Lafontaine (1998, 2004). Both Jones’ (1914) and Schaus’ (1914) descriptions were thorough, although for the species treated here devoted primarily to wing pattern. Rather that reproduce them, we provide instead descriptions of the genitalia and other characters of note as well as formal diagnoses.

The male clasping apparatus differs significantly from that of *Leucosigma* Druce, and our terminology warrants clarification supplementary to that in the parallel review of that genus (Goldstein et al. 2018, this issue). We call specific attention to characters enumerated below in the generic re-description and in Figs 91–99. Although analogous in location, these structures do not all correspond to numbered structures referenced in our discussion of *Leucosigma*.

Provisional (neighbor-joining) analyses of available DNA barcode data helped to guide dissection efforts and taxonomic decisions, and were supplemented by a partial sequence (~562bp) extracted during the dissection of the type of *Lophomyrasantista*. That extraction involved an overnight soak of the abdomen in proteinase and sequestration of the lysate prior to soaking the abdomen in KOH per the normal dissection protocol. It is hoped that a more thorough treatment of *Lophomyra* will be enabled by greater availability of specimens and corresponding sequence data.

### Repository abbreviations

The following abbreviations refer to collections from which specimens form the basis of this work:

**MNHUK**The Natural History Museum, London, UK (formerly BMNH).

**USNM**National Museum of Natural History [formerly, United States National Museum], Washington, District of Columbia, USA.

## Systematics

### Lophomyra

Taxon classificationAnimaliaLepidopteraNoctuidae

Schaus, 1911

#### Synonym.

*Iheringia* Jones, [1915] 1914.

#### Type species.

*Iheringiasantista* Jones, 1908 by monotypy.

#### Diagnosis.

Species of *Lophomyra* are most unmistakably diagnosed by the male genitalia, specifically a conspicuous uncus that *in situ* appears swollen with a silvery or dark-gray sheen of scale-like clusters of setae, each cluster sharing a setal socket and shingled (hence scale-like). Forewing variously shaded with moss-green scales; paler scaling, to the extent present, concentrated primarily towards the inner margin; sexual dimorphism discernible with females more darkly colored, their hind wings more uniformly dark gray throughout and forewing pattern elements generally more distinct than in males; males bear a pronounced dorsal tuft on the second abdominal segment.

#### Description.

**Head.** Labial palpi, frons, and vertex scaled with a mixture of whitish, gray brown, and green. Labial palpi upturned, with second segment longer than the other two segments combined. Proboscis with paired lateral rows of small protuberances towards terminus. Eyes smooth. Antennae filiform, finely scaled dorsally with white or a mixture of white and green.

**Thorax.** Vestiture predominantly made up of spatulate scales and simple hairs; a mix of gray-brown, purplish, lime-green scaling and black peppering; paired latero-dorsal tufts of elongate hairs arising at base of metathorax towards abdomen. *Wings*. Forewing a mix of gray-brown, black, lime- and moss-green, white and cream-colored scales, the green most prominent in the medial and terminal areas and basally along the inner margin; lines generally incomplete, the black postmedial line most visible but broken, bordered with white on both sides; medial veins edged in black towards the outer margin. Basal, antemedial and medial costal striae black and white; postmedial striae white only. Pattern element boundaries blurred in part by variably shaded scaling and most particularly by medial streaking in *L.tacita* and *L.santista*. *Legs*. Scaled with a mixture of green and white; a single pair of mid-tibial spurs; two pair on hind-tibiae; 2+ rows of tibial spines on foreleg, three rows on mid- and hind-legs.

**Abdomen.** Scales predominantly grayish tan. Dorsal tuft of brown scales on second abdominal segment of males. Green scales intermixed with grayish tan, darkening towards in terminal segments. Note the complex of dorsal and ventral tufts enclosing and subtending the uncus and valvae, respectively.

**Male genitalia.** Uncus shingled with dark gray or silvery scale-like setal clusters, each representing ~7–9 spine-like setae. Saccus blunt. Juxta roughly shield-shaped, the dorsal edge concave, slightly jagged. Each divided valva comprises (1) a weakly sclerotized elongate sacculus; (2) a strongly sclerotized clasper, either a rudimentary beaklike structure or an elongate gently curved and concave spike, fused to the cucullus (3), which is elongate, weakly sclerotized, and may be swollen apically, spatulate or club-like with a heavy covering of setae and slightly recurved, such that the pair of these structures flank the uncus; and (4) a minor, ampulla-like process embedded within the sacculus, with which it may form a crotch that cradles the clasper. Directly beneath this structure on the inner face of the valve is what appears a well-developed editum comprising a raised patch of spine-like setae or, in the larger species, at least 10 fully developed spines directed anteromedially. Vesica unadorned, without cornuti, but a bilobate sub-basal diverticulum, highly bulbous in the larger species.

**Female genitalia.** Papillae anales blunt-tipped, subquadrate; ductus bursae and corpus bursae flask or wineskin shaped, colliculum absent; ductus bursae narrow relative to caudal region of bursa, constricted at (dorsal) juncture of the two, opposite dorsal opening to ductus seminalis; ductus may have small ventral posterior appendicular lobe; corpus bursae without signa.

**Immature stages.** Larvae, known exclusively from images, have predominantly orange or reddish-orange heads with 10 black spots, and paired setose dorsal spines.

#### Key to known species of *Lophomyra*

**Table:** 

1	Forewing with a mottled appearance overall, but markings generally distinct, including white-outlined figure 8-shaped reniform and orbicular spots and a black spot at the inner margin near the forewing base; forewing margin prominently scalloped; antrum broadly V-shaped, its sides directed away from one another, not parallel; ductus bursae with small dorsal appendicular lobe	*** L. commixta ***
–	Forewing with a smudged appearance overall, markings variably distinct; reniform and orbicular spots figure 8-shaped but outlined faintly, if at all; inner edge of forewing smooth gray or grayish green; forewing margin not conspicuously scalloped; antrum chalice-shaped, its sides parallel or nearly so; ductus bursae with or without small dorsal appendicular lobe	**2**
2	Forewing with almost no black markings; reniform not outlined in white, appearing as a brown smudge; ductus bursae with small dorsal appendicular lobe	*** L. santista ***
–	White-outlined figure 8-shaped reniform spot; black basal dash extends almost to antemedial line; ductus bursae without small dorsal appendicular lobe	*** L. tacita ***

### Lophomyra commixta

Taxon classificationAnimaliaLepidopteraNoctuidae

(Schaus, 1914) comb. n.

Figs 49–56 , 57–64 ; Male genitalia: Figs 69–72 , 81–84 ; Figs 95 , 98 , 99 ). Female genitalia: Figs 85 , 86 ; Larvae: Figs 100–107

Chytonidia commixta Schaus, 1914. Type locality: French Guiana.

#### Material examined.

**Type material**. HOLOTYPE ♂: FRENCH GUIANA: St. Laurent, Maroni, *Chytonixcommixta* Type Schs, Collection Wm Schaus, Type No. 16530 U.S.N.M., USNMENT01370304, ♂USNM Dissection 148186. Type at USNM.

#### Other material examined.

(11♂, 14♀) **FRENCH GUIANA** (2♂, 5♀): ♂AOUT, GUYANE FRANÇAISE St-LAURENT du MARONI COLL LE MOULT, Dognin Collection, USNMENT01437236, Male genitalia imaged *in situ*; ♂ JUILLET, Ibid, USNMENT01438844, USNM Dissection 148087; ♀JUILLET, Ibid, USNMENT01437245; ♀ JANVIER, Ibid, USNMENT01437277; ♀ Cayenne, F. Guiana., Collection Wm Schaus, USNM Dissection 148086, USNMENT01370319; ♀Ibid, *Chytonixcommixta* Schs., USNMENT01370317; ♀ S.-Laurent du Maroni Guy. Franc, Dognin Collection, *Chytonixcommixta* Schs., USNMENT01370327 **COSTA RICA** (9♂, 9♀):

The following label data precede Santa Rosa National Park (SRNP) identifier codes on all reared and light-trapped specimens examined (16♂, 29♀): Voucher: D.H. Janzen & W. Hallwachs DB: http://janzen.sas.upenn.edu Area de Conservacion Guanacaste, COSTA RICA. Except for those denoted “Alajuela,” all localities are within Guanacaste Province.

*Males*: Alajuela: Sector Rincon Rain Forest: Jacobo, 10.94076, -85.3177, el. 461m: larva on *Microgrammapercussa*: 01/07/2011, ecl. 02/03/2011, Edwin Apu, collector, 11-SRNP-69041, USNMENT01370325, USNM Dissection 148098; Sector Pitilla: Estacion Quica, 10.99697, -85.39666, el. 470m: larva on *Microgrammapercussa*: 01/27/2010, ecl. 03/05/2010, Ricardo Calero, collector, 10-SRNP-70532, USNMENT01438818; Sector Pitilla: Estacion Quica, 10.99697, -85.39666, el. 470m: larva on *Microgrammapercussa*: 01/27/2010, ecl. 03/06/2010, Ricardo Calero, collector, 10-SRNP-70533, USNMENT01438849; Sector Pitilla: Estacion Quica, 10.99697, -85.39666, el. 470m: larva on *Polypodiumfraxinifolium*: 01/29/2010, ecl. 03/06/2010, Manuel Rios, collector, 10-SRNP-70574, USNMENT01437246; Sector Pitilla: Estacion Quica, 10.99697, -85.39666, el. 470m: larva on *Polypodiumfraxinifolium*: 01/29/2010, ecl. 03/05/2010, Manuel Rios, collector, 10-SRNP-70573, USNMENT01437262; Sector Pitilla: Estacion Quica, 10.99697, -85.39666, el. 470m: larva on *Microgrammapercussa*: 01/07/2014, ecl. 02/17/2014, Ricardo Calero, collector, 14-SRNP-70035, USNMENT01438824; Sector Pitilla: Estacion Quica, 10.99697, -85.39666, el. 470m: larva on *Niphidiumoblanceolatum*: 01/25/2010, ecl. 02/27/2010, Ricardo Calero, collector, 10-SRNP-70488, USNMENT01438848, USNM Dissection 148180; Sector Pitilla: Estacion Quica, 10.99697, -85.39666, el. 470m: larva on *Microgrammapercussa*: 02/15/2010, ecl. 03/24/2010, Ricardo Calero, collector, 10-SRNP-70810, USNMENT01370328, USNM Dissection 148050; Sector Pitilla: Estacion Quica, 10.99697, -85.39666, el. 470m: larva on *Microgrammapercussa*: 02/15/2010, ecl. 03/20/2010, Dinia Martinez, collector, 10-SRNP-70812, USNMENT01437227.

*Females*: Sector Pitilla: Estacion Quica, 10.99697, -85.39666, el. 470m: larva on *Niphidiumoblanceolatum*: 01/21/2010, ecl. 03/04/2010, Ricardo Calero, collector, 10-SRNP-70495, USNMENT01437182; Sector Pitilla: Estacion Quica, 10.99697, -85.39666, el. 470m: larva on *Microgrammapercussa*: 01/24/2010, ecl. 03/02/2010, Ricardo Calero, collector, 10-SRNP-70470, USNMENT01437196, USNM Dissection 148299; Sector Pitilla: Estacion Quica, 10.99697, -85.39666, el. 470m: larva on *Polypodiumfraxinifolium*: 02/03/2010, ecl. 03/17/2010, Ricardo Calero, collector, 10-SRNP-70609, USNMENT01437192; Sector Pitilla: Estacion Quica, 10.99697, -85.39666, el. 470m: larva on *Microgrammapercussa*: 11/07/2010, ecl. 12/22/2010, Ricardo Calero, collector, 10-SRNP-73249, USNMENT01437207; Sector Pitilla: Estacion Quica, 10.99697, -85.39666, el. 470m: larva on *Polypodiumfraxinifolium*: 01/29/2010, ecl. 03/11/2010, Manuel Rios, collector, 10-SRNP-70572, USNMENT01437261; Sector Pitilla: Estacion Quica, 10.99697, -85.39666, el. 470m: larva on *Microgrammapercussa*: 02/15/2010, ecl. 03/24/2010, Dinia Martinez, collector, 10-SRNP-70811, USNMENT01437202; Sector Pitilla: Estacion Quica, 10.99697, -85.39666, el. 470m: larva on *Polypodiumfraxinifolium*: 01/27/2010, ecl. 03/03/2010, Dinia Martinez, collector, 10-SRNP-70534, USNMENT01437267 USNM Dissection 148187; Sector Pitilla: Estacion Quica, 10.99697, -85.39666, el. 470m: larva on *Microgrammapercussa*: 01/23/2010, ecl. 03/02/2010, Ricardo Calero, collector, 10-SRNP-70436, USNMENT01370329, USNM Dissection 148051; Sector Pitilla: Estacion Quica, 10.99697, -85.39666, el. 470m: larva on *Microgrammapercussa*: 03/15/2012, ecl. 04/18/2012, Ricardo Calero, collector, 12-SRNP-70644, USNMENT01370326, USNM Dissection 148106.

#### Diagnosis.

Smaller and with distinct patterning and genitalic differences that distinguish it from the other described *Lophomyra*. Overall, we note the more mottled appearance to the forewing than in *L.tacita* or *L.santista*. More specifically, the green orbicular spot outlined in cream and the reniform spot outlined in white form a pair of figure 8’s in *L.commixta* that meet at their posterior edges to form a deformed U-shaped stigma. These pattern elements are less obvious in *L.tacita* and *L.santista*, where the fusion of the reniform and orbicular spots is more complete. The male genitalia of *L.commixta* are distinct from and less robust than those of its congeners by virtue of the clasper’s being less sharply developed, and the cucullus' appearing simple and spatulate; the vesica bears a basal diverticular nipple (Figs 81–84). Female *L.commixta* also bear an appendicular lobe at the caudal end of the ductus bursae, a feature shared with *L.santista* but not with *L.tacita*.

#### Re-description.

**Head**. Labial palpi as for genus. Proboscis with paired lateral rows of ~28 small protuberances at terminus. Frons and vertex scaled with a mixture of whitish and grayish brown. Remaining scales of head and palpi a mixture of white, gray-brown and “lilacine” (cf. Schaus 1914: 487). Antennae filiform, finely scaled, dorsally edged with white and a few green scales toward the base.

**Thorax**. Male prothorax with two fans of stalked scales, predominantly lime green, each fan gray-brown at its center and peppered with black; a third predominantly brown medial crest immediately posterior. *Wings.* Forewing length 11.5 mm (holotype, male), average 10.6 mm (males, *n* = 9), 11.5 mm (females, *n* = 7). Forewing patterning appearing less blended than in congeners, largely due to visibility of reniform-subreniform complex (see above) and the visibility of the postmedial line; on undersides, green shading confined primarily to forewing terminal areas; post-medial lines present but faint on undersides. *Legs.* Scaling, tibial spurs, and rows of tibial spines as for genus.

**Abdomen**. Dorsal scales predominantly tannish, except on terminal segments where visibly green; a medial line of dark scaling ventrally. Males with prominent medio-dorsal tuft of brown spatulate scales on A2; dorsal tufts posterior to A2 composed primarily of hairs concolorous with adjacent abdominal scaling.

**Male genitalia**. Structures less robust than those of larger congeners, including the much-reduced sacculus (1), a barely visible ampulla-like structure within it (4); the spines associated with the editum; the clasper (2), which is small and beak-like; and the cucullus (3), which is spatulate or modestly swollen distally, and not strongly recurved. Vesica with a shallow subbasal diverticular bump and a separate, more conspicuous, basal nipple.

**Female genitalia**. Antrum narrow, not more than twice the width of the ductus; ventral appendicular lobe present at caudal end of ductus bursae.

**Immature stages**. Larvae known from images (Figs 100–107). Pattern highly disruptive, with less contrast in coloration amid thoracic than abdominal segments. Head capsule pale with reddish calico patterning and black pinacula; dorsal chalazae red and raised, bearing bicolorous pinacula, black towards the front, red backward, forming setose spines, most prominent on A1–3; cream-colored subdorsal patches lateral to each spine form a series of ventro-caudally directed diagonal streaks, faintly shaded with green, again most conspicuous on A1–3, fainter on A4–7 and stronger again on A8; highly anastomosing pattern of fine pale lines centered mid-dorsally. This color pattern is highly cryptic among tangles of fern rhizomes where the caterpillars rest and pupate.

#### Biology.

Wild-caught caterpillars were found feeding on leaves of *Microgrammapercussa*, *Niphidiumoblanceolatum*, *Polypodiumfraxinifolium* (all Polypodiaceae). Eighteen reared individuals (10 males, 8 females) used an average of 24 days between the onset of the prepupal stage and adult eclosion in their ACG rain forest habitat. All ACG specimens were reared from wild-caught caterpillars and none light-trapped despite massive ACG-wide light-trapping, and all have the same DNA COI barcode and BIN (BOLD:AAY4740). No barcodes were available for South American specimens.

#### Distribution.

French Guiana, Costa Rican rain forest.

#### Remarks.

The conspicuous genitalic similarities as well as provisional analyses of DNA barcode data corroborate the placement of “*Chytonidia*” *commixta* with *Lophomyra*. The reared Costa Rican specimens may well represent a species distinct from *L.commixta*; they are larger than a small series of topotypic specimens from French Guiana (and reared specimens are commonly smaller than wild-caught adults). However, in the absence of evidence to the contrary, we have elected to continue to include them under *L.commixta*, recognizing that additional data may well separate the two, and that it is not un common for South American specimens to be recognized as taxonomically distinct from their Costa Rican look-alikes (e.g., Grishin et al. 2013, Janzen et al. 2017).

### Lophomyra tacita

Taxon classificationAnimaliaLepidopteraNoctuidae

Schaus, 1911

Figs 1–10 , 11–20 , 21–30 , 31–40 ; Male genitalia: Figs 65–68 , 73–76 , 91 , 92 , 93 , 94 , 96 , 97 ; Female genitalia: Figs 88–90 ; Larvae: Figs 108–115 ; Pupae: Figs 116 , 117–118

Lophomyra tacita Schaus, 1911 Type locality: Costa Rica.

#### Material examined.

**Type material**. HOLOTYPE ♀: Mar, Sixola Riv CR, Type No. 17334 U.S.N.M., *Lophomyratacita* Type Schs, USNMENT00973292, USNM Dissection 148201. Types at USNM.

#### Other material examined.

(22♂, 30♀) **VENEZUELA** (2♂,1♀): VENEZUELA: Aragua Rancho Grande 1100m 1–3 IV 1978 blacklight, cloud forest, J. B. Heppner, USNM Dissection 148083, USNMENT01437226 [♂]; VENEZUELA: Ar. Rancho Grande July1–7 1967 RW Poole 1100m, USNMENT01370311 [♂]; Ibid July 15–21, USNM Dissection 148084, USNMENT01370316 [♀].

**COSTA RICA** (19♂, 29♀): ♂Juan Vinas CR, June, *Lophomyratacita* Schs, gen & sp nov *Xylomyges* group USNMENT01370323; Turrialba Costa Rica 10 II 1973 V.O. Becker, col. Becker 33420, *Lophomyratacita* Schs, 1911, USNM Dissection 148143, USNMENT01370321; Ibid, USNM Dissection 148081, USNMENT01438834.

The following label data precede SRNP identifier codes on all reared and light-trapped specimens examined (16♂, 29♀): Voucher: D.H. Janzen & W. Hallwachs DB: http://janzen.sas.upenn.edu Area de Conservacion Guanacaste, COSTA RICA. Except for those denoted “Alajuela”, all localities are within Guanacaste Province.

*Males* (16): Sector Pitilla: Estacion Quica, 10.99697, -85.39666, el. 470m: larva on *Microgrammapercussa*: 11/29/2009, ecl. 01/05/2010, Dinia Martinez, collector, 09-SRNP-73925, USNMENT01370314, USNM Dissection 148052; Sector Pitilla: Quebradona, 10.99102, -85.39539, el. 475m: larva on *Microgrammapercussa*: 01/10/2010, ecl. 03/02/2010, Ricardo Calero, collector, 10-SRNP-70181, USNMENT01437251, USNM Dissection 148188; Sector Pitilla: Quebradona, 10.99102, -85.39539, el. 475m: larva on *Pleopeltispolypodioides*: 05/03/2011, ecl. 05/29/2011, Petrona Rios, collector, 11-SRNP-70989, USNMENT01370310; Sector Pitilla: Estacion Quica, 10.99697, -85.39666, el. 470m: larva on *Niphidiumoblanceolatum*: 11/21/2010, ecl. 12/28/2010, Ricardo Calero, collector, 10-SRNP-73286, USNMENT01370301; Sector Pitilla: Estacion Quica, 10.99697, -85.39666, el. 470m: larva on *Microgrammapercussa*: 10/21/2010, ecl., Ricardo Calero, collector, 10-SRNP-73135, USNMENT01438813; Sector Pitilla: Estacion Quica, 10.99697, -85.39666, el. 470m: larva on *Microgrammapercussa*: 10/21/2010, ecl. , Ricardo Calero, collector, 10-SRNP-70435, USNMENT01437276, USNM Dissection 148202; Sector Pitilla: Estacion Quica, 10.99697, -85.39666, el. 470m: larva on *Microgrammapercussa*: 11/29/2009, ecl. 01/01/2010, Ricardo Calero, collector, 09-SRNP-73965, USNMENT01438858; Sector Pitilla: Estacion Quica, 10.99697, -85.39666, el. 470m: larva on *Polypodiumfraxinifolium*: 02/09/2010, ecl. 03/20/2010, Dinia Martinez, collector, 10-SRNP-70707, USNMENT01370287; Sector Pitilla: Estacion Quica, 10.99697, -85.39666, el. 470m: larva on *Polypodiumfraxinifolium*: 01/29/2010, ecl. 03/11/2010, Manuel Rios, collector, 10-SRNP-70571, USNMENT01437222; Sector Pitilla: Calma, 11.00987, -85.39214, el. 412m: larva on *Microgrammapercussa*: 01/29/2010, ecl. 03/13/2010, Ricardo Calero, collector, 10-SRNP-70569, USNMENT01437252; Sector Pitilla: Estacion Quica, 10.99697, -85.39666, el. 470m: larva on *Microgrammapercussa*: 10/21/2010, ecl. 11/20/2010, Ricardo Calero, collector, 10-SRNP-73128, USNMENT01370305; Sector Pitilla: Estacion Quica, 10.99697, -85.39666, el. 470m: larva on *Microgrammapercussa*: 02/01/2010, ecl. 03/10/2010, Ricardo Calero, collector, 10-SRNP-70597, USNMENT01438869; Sector Pitilla: Estacion Quica, 10.99697, -85.39666, el. 470m: larva on *Microgrammapercussa*: 02/08/2010, ecl. 03/13/2010, Ricardo Calero, collector, 10-SRNP-70719, USNMENT01438854; Sector Pitilla: Estacion Quica, 10.99697, -85.39666, el. 470m: larva on *Polypodiumfraxinifolium*: 01/26/2010, ecl. 03/02/2010, Ricardo Calero, collector, 10-SRNP-70530, USNMENT01438809; Sector Pitilla: Estacion Quica, 10.99697, -85.39666, el. 470m: larva on *Polypodiumfraxinifolium*: 11/26/2010, ecl. 12/03/2010, Dinia Martinez, collector, 10-SRNP-73310, USNMENT01437265, USNM Dissection 148099; Sector Pitilla: Estacion Quica, 10.99697, -85.39666, el. 470m: larva on *Microgrammapercussa*: 10/30/2010, ecl. , Dinia Martinez, collector, 10-SRNP-73207, USNMENT01370315.

*Females* (29): ♀ Costa Rica. Juan Vinas. 2500 ft. June Wm Schaus 1911-32, *Loxodestacita* Schs, NHMUK01606199; Alajuela: Sector San Cristobal: Finca San Gabriel, 10.87766, -85.39343, el. 645m: larva on *Campyloneurumgracile*: 03/19/2012, ecl. 04/20/2012, Elda Araya, collector, 12-SRNP-1080, USNMENT01370309; Alajuela: Sector San Cristobal: Estacion San Gerardo, 10.88009, -85.38887, el. 575m: 11/21/2006, F.Quesada&H.Cambronero, collector, 06-SRNP-109388, USNMENT01437195; Alajuela: Sector San Cristobal: Estacion San Gerardo, 10.88009, -85.38887, el. 575m: 05/04/2011, R.Franco&S.Rios, collector, 11-SRNP-103237, USNMENT01437212; Alajuela: Sector Rincon Rain Forest: Jacobo, 10.94076, -85.3177, el. 461m: larva on *Microgrammapercussa*: 01/11/2011, ecl. 02/09/2011, Edwin Apu, collector, 11-SRNP-69085, USNMENT01437180; Sector Pitilla: Estacion Quica, 10.99697, -85.39666, el. 470m: larva on *Microgrammapercussa*: 01/19/2010, ecl. 02/23/2010, Dinia Martinez, collector, 10-SRNP-70396, USNMENT01437191, USNM Dissection 148181; Sector Pitilla: Estacion Quica, 10.99697, -85.39666, el. 470m: larva on *Polypodiumfraxinifolium*: 02/02/2010, ecl. 03/04/2010, Ricardo Calero, collector, 10-SRNP-70590, USNMENT01370320, USNM Dissection148189; Sector Pitilla: Coneja, 11.01525, -85.39766, el. 415m: larva on *Microgrammapercussa*: 06/24/2010, ecl. 07/29/2010, Ricardo Calero, collector, 10-SRNP-71914, USNMENT01370306; Sector Pitilla: Estacion Quica, 10.99697, -85.39666, el. 470m: larva on *Polypodiumfraxinifolium*: 02/09/2010, ecl. 03/17/2010, Dinia Martinez, collector, 10-SRNP-70706, USNMENT01370302; Sector Pitilla: Estacion Quica, 10.99697, -85.39666, el. 470m: larva on *Microgrammapercussa*: 10/30/2010, ecl. , Dinia Martinez, collector, 10-SRNP-73205, USNMENT01437237; Sector Pitilla: Estacion Quica, 10.99697, -85.39666, el. 470m: larva on *Microgrammapercussa*: 08/17/2010, ecl. 09/24/2010, Ricardo Calero, collector, 10-SRNP-72638, USNMENT01370313, USNM Dissection 148145; Sector Pitilla: Estacion Quica, 10.99697, -85.39666, el. 470m: larva on *Microgrammapercussa*: 03/26/2011, ecl. 04/26/2011, Dinia Martinez, collector, 10-SRNP-70741, USNMENT01437210, USNM Dissection 148144; Sector Pitilla: Estacion Quica, 10.99697, -85.39666, el. 470m: larva on *Niphidiumoblanceolatum*: 10/21/2010, ecl. 11/30/2010, Ricardo Calero, collector, 10-SRNP-73136, USNMENT01437215; Sector Pitilla: Estacion Quica, 10.99697, -85.39666, el. 470m: larva on *Microgrammapercussa*: 01/23/2010, ecl. 03/06/2010, Ricardo Calero, collector, 10-SRNP-70437, USNMENT01437235; Sector Pitilla: Estacion Quica, 10.99697, -85.39666, el. 470m: larva on *Microgrammapercussa*: 11/03/2010, ecl. , Ricardo Calero, collector, 10-SRNP-73225, USNMENT01370322; Sector Pitilla: Estacion Quica, 10.99697, -85.39666, el. 470m: larva on *Microgrammapercussa*: 02/26/2010, ecl. 04/01/2010, Ricardo Calero, collector, 10-SRNP-70954, USNMENT01437190; Sector Pitilla: Estacion Quica, 10.99697, -85.39666, el. 470m: larva on *Microgrammapercussa*: 01/19/2010, ecl. 02/23/2010, Ricardo Calero, collector, 10-SRNP-70395, USNMENT01437256; Estacion Quica, 10.99697, -85.39666, el. 470m: larva on *Microgrammapercussa*: 01/26/2010, ecl. 03/15/2010, Manuel Rios, collector, 10-SRNP-70493, USNMENT01438828; Sector Cacao: Roca Verde, 10.89354, -85.43603, el. 835m: 08/12/2007, R.Franco&F.Quesada, collector, 07-SRNP-108035, USNMENT01437232; Sector Pitilla: Estacion Quica, 10.99697, -85.39666, el. 470m: larva on *Microgrammapercussa*: 11/27/2013, ecl. 01/05/2014, Ricardo Calero, collector, 13-SRNP-71895, USNMENT01370324; Sector Pitilla: Estacion Quica, 10.99697, -85.39666, el. 470m: larva on *Microgrammapercussa*: 10/08/2013, ecl. 11/11/2013, Ricardo Calero, collector, 13-SRNP-71692, USNMENT01437185; Sector Pitilla: Estacion Quica, 10.99697, -85.39666, el. 470m: larva on *Microgrammapercussa*: 12/04/2010, ecl. 01/17/2011, Ricardo Calero, collector, 10-SRNP-73361, USNMENT01437205; Sector Pitilla: Estacion Quica, 10.99697, -85.39666, el. 470m: larva on *Microgrammapercussa*: 11/07/2009, ecl. 12/10/2009, Dinia Martinez, collector, 09-SRNP-73662, USNMENT01437270; Sector Pitilla: Estacion Quica, 10.99697, -85.39666, el. 470m: larva on *Microgrammapercussa*: 01/06/2010, ecl. 02/19/2010, Ricardo Calero, collector, 10-SRNP-70072, USNMENT01370280; Sector Pitilla: Calma, 11.00987, -85.39214, el. 412m: larva on *Niphidiumoblanceolatum*: 02/22/2011, ecl. 04/11/2011, Ricardo Calero, collector, 11-SRNP-70494, USNMENT01370312; Sector Pitilla: Estacion Quica, 10.99697, -85.39666, el. 470m: larva on *Polypodiumfraxinifolium*: 01/26/2010, ecl. 03/05/2010, Manuel Rios, collector, 10-SRNP-70496, USNMENT01370308; Sector Pitilla: Amonias, 11.04249, -85.40339, el. 390m: larva on *Microgrammapercussa*: 08/21/2010, ecl. 09/19/2010, Manuel Rios, collector, 10-SRNP-31891, USNMENT01438853; Sector Pitilla: Coneja, 11.01525, -85.39766, el. 415m: larva on *Microgrammapercussa*: 01/23/2010, ecl. 03/03/2010, Ricardo Calero, collector, 10-SRNP-70460, USNMENT01437275, USNM Dissection 148053; Sector Pitilla: Estacion Quica, 10.99697, -85.39666, el. 470m: larva on *Microgrammapercussa*: 11/27/2013, ecl. 01/05/2014, Ricardo Calero, collector, 13-SRNP-71894, USNMENT01438803, USNM Dissection 148105; Sector Pitilla: Estacion Quica, 10.99697, -85.39666, el. 470m: larva on *Microgrammapercussa*: 07/02/2010, ecl. 08/07/2010, Ricardo Calero, collector, 10-SRNP-71962, USNMENT01437216.

#### Diagnosis.

Readily distinguished from *L.commixta* as both larva and adult; adult more similar to that of *L.santista* (below) but can be distinguished by white costal frosting on distal half of forewing, the interruption of the U-shaped stigma by a thin longitudinal white streak which partially encircles a black dot at the base of the reniform, and a variable diffuse gray patch between the dash and a more expansive gray-green patch along the inner margin near the base of the wing. Females lack the appendicular lobe on the ductus bursae, present in both the other species.

#### Re-description.

**Head**. Labial palpus with 2^nd^ segment >2× combined length of first and third combined; all segments scaled with a mixture of whitish, black, brown and green. Proboscis with paired lateral rows of ~28 small protuberances at terminus. Eyes smooth. Antennae filiform, dorsally with fine white scales; frons, vertex, and palpi (all segments) with a mix of white and green scales.

**Thorax**. Thoracic vestiture a mix of green, white, black, and light brown scales. *Wings.* Forewing length 13.3 mm (holotype, female), average 12.7 mm (males, *n* = 9), 13.2 mm (females, *n* = 11). Forewing patterned with gray, white, black and moss-green scaling, the last predominantly in the subcostal, outer medial, and subterminal areas and along inner margin; basal line confined to a black subcostal spot or (in females) a pair of black subcostal spots; the most extensive green scaling forming a uniform green basal patch along the inner margin between CuA2 and the postmedial line; short, black basal dash black along inner edge of M, forming the leading edge of a purplish-gray wedge; an outer medial wedge, predominantly moss green, between CuA1 and M2, the latter of which is also edged in purplish gray, overlaps with the fusion of the reniform and orbicular spots; subterminal line wavy, shadowed by white scaling and punctuated by black dots at the intersections of each vein; orbicular spot elongate, lime green, outlined in gold scaling, converging below the M vein with dumbbell-shaped, moss-green reniform spot, outlined with lime-green scaling, forming a deformed U-shape; white subcostal frosting along distal half of wing; a pale, washed-out patch in the ventro-posterior part of the wing, most conspicuous in males. Hind wing uniformly gray in females, pale basally in males, with discal spot faint but present in both. Underside of forewing terminal area with noticeably green shading; inner margin pale; center of wing gray, paler towards inner margin. Pattern elements on underside less conspicuous in males than females, visible primarily in the terminal area of the forewing and the costal margin of the hind wing, where the discal spot is likewise faint, if present in males, and the postmedial line wavy, brown, outwardly white in costal part and increasingly diffuse towards the inner margin. *Legs.* Scales primarily a mixture of green and white; tibial spurs and rows of tibial spines as for genus.

**Abdomen**. Tan above; abdominal segments with medio-dorsal tufts of tan spatulate scales, tipped brown on A1 and A2, A2 the most prominent, decreasing in size from A3–5; each abdominal segment ringed with an apical ring of elongate, strap-like scales and a basal ring of hairs; medial paired medial tufts of green scales ventrally; terminal tufts elaborate, one pair of lateral tufts arcing medially over uncus, when exposed; recurved, tufted apices of dorsal process of cucullus may direct outwardly when viewed *in situ* (Figs 93, 96, 97).

**Male genitalia**. Uncus, tegumen, vinculum, and saccus as for genus. Juxta U-shaped, the medio-lateral edges jagged. Sacculus (1) finger-like. Clasper (2) pronouncedly sickle-like and concave. Cucullus (3) especially robust, heavily setose, club-like and recurved apically. Ampulla-like structure (4) embedded within sacculus anterior to a prominent patch of medially directed spines. Vesica with bilobate diverticulum encircling its base.

**Female genitalia**. Appendicular lobe absent from ductus bursae, which is elongate, >⅓ the length of the corpus bursae; antrum wide and well developed.

**Immature stages**. Larvae known from images (Figs 108–115). Overall coloration orange and black, Cephalic spots larger and somatic chalazae more developed and sharper than in *L.commixta*, the latter black throughout and bearing two setae apiece. Head capsule orange with five black spots on each side: dorsal/vertical, posterio-dorsal, anterior, lateral, and substemmatal (genal). Frons black; antennae orange at base, otherwise black. Dorsum tapers downward from A1 to T3 as in *L.commixta*. Chalazae black, raised, the dorsal pairs forming robust bisetose spines most prominent on the thoracic and anterior abdominal segments. Integument orange, dorsum spotted black thoracically, thereafter a combination of linear black dashes and black stripes originating dorsally on either side of the orange mid-dorsal line at the caudal end of each segment, giving rise to a striped “herring-bone” pattern and rendering the appearance of bifurcating orange stripes extending forward and down to form a series of slanting alternating black and orange stripes; the orange subspiracular line becoming cantaloupe orange and broader in later instars.

#### Biology.

Larvae found feeding on leaves of *Microgrammapercussa, Niphidiumoblanceolatum, Polypodiumfraxinifolium, Pleopeltispolypodioides, Campyloneurumgracile* (all Polypodiaceae) in ACG rainforest. Thirty-five individuals (11 males, 24 females) took an average of 25 days between the observed onset of the prepupal stage and adult eclosion, with ranges of 21–31 days for males and 19–29 days for females. These data are all from their ACG rain forest habitat. Almost all ACG specimens were reared from wild-caught caterpillars and only three light trapped despite massive ACG-wide light trapping, and all have the same DNA COI barcode and BIN (BOLD:AAJ2401). No barcodes were available for other specimens of *L.tacita*.

#### Distribution.

Costa Rica, Venezuela.

### Lophomyra santista

Taxon classificationAnimaliaLepidopteraNoctuidae

(Jones, [1915] 1914)

Figs 41–48 ; Male genitalia: Figs 70 , 80 ; Female genitalia: Fig. 87

Iheringia santista Jones, (1915) 1914 Type locality: Brazil.

#### Material examined.

**Type material** (2♂). SYNTYPES: [Brazil] Type, *Iheringiasantista* type ♂ Jones, Alto da Serra Santos 800m. 25 Feb. 1913 E.D. Jones, NHMUK01606195; Alto da Serra Santos 800m. 9 Mar. 1913, E.D. Jones coll., Brit. Mus. 1919-295., NHMUK01606197. Types at NMHUK.

#### Other material examined

(3♂, 1♀). **BRAZIL** (2♂): BRAZIL: Santa Catharina. Blumenau. Neu Bremen. 28 VIII. 1932, Fritz Hoffmann. B.M. 1934-63, NHMUK01606196; Alto de Serra, Sao Paulo February, 1933. (R. Spitz), Rothschild Bequest B.M. 1939-1., NHMUK01606198. **FRENCH GUIANA** (1♂, 1♀): Juin, Guyane Franc^se^ Nouveau Chantier Collection Le Moult, *Lophomyratacita* Schs [illeg.] 6-2-13, Dognin Collection, USNMENT01438868; St. Jean, Maroni, F. Guiana. Collection Wm Schaus, *Lophomyratacita* Schs, USNM Dissection 148082, USNMENT01370318.

#### Diagnosis.

Two elongate chocolate brown patches towards base of forewing; apical spot less pronouned than in *L.tacita*; ventral hind wing with postmedial line less pronounced than in *tacita*; male genitalia nearly indistinguishable from those of *L.tacita* but quite distinct from *L.commixta*; unlike *L.tacita*, female genitalia bear the appendicular lobe at caudal end of ductus bursae.

#### Re-description.

**Head.** Antennae filiform, dorsally with fine white scales; eyes naked; scaling on vertex, frons and palpi much like that of *L.tacita* but greenish scaling faded in available material so direct comparisions difficult except to Costa Rican *L.tacita*.

**Thorax**. Vestiture similar to that of head. *Wings.* Forewing length 14.4 mm (holotype, male), average 14.3 mm (males, *n* = 4), 13.9 mm (female, *n* = 1). Underside of forewing variably frosted along costal and outer margins, whitish along inner margin, uniformly pale gray throughout outer part of wing; hind wing underside pale inward, gray-brown and white dusting along costal margin; discal spot faint. *Legs.* Scaling, tibial spurs, and rows of tibial spines as for genus.

**Abdomen**. Although Jones (1915: 440) describes both the thorax and abdomen as “without crests” in the generic description of the monotypic *Iheringia*, of which *L.santista* is the type, there are concolorous dorsal tufts on the first 8 abdominal segments of males of this species and *L.tacita*.

**Male gentialia**. Structures, including vesica, not readily distinguishable from those of *L.tacita*.

**Female genitalia**. Based on a single French Guiana specimen (Fig. 87), comparable to *L.tacita* except for the presence of ventral appendicular lobe on the caudal end of the ductus bursae. This specimen appears particularly distended because it contains three spermatophores.

**Immature stages**. Unknown.

#### Biology.

Unknown.

#### Distribution.

Brazil and French Guiana.

#### Remarks.

Available barcode data were supplemented by a partial sequence of the holotype of *L.santista*, but were not, in our estimation, sufficient for unambiguous diagnosis or synonymy with *L.tacita*, particularly given that the primary types of *L.santista* are male, and that of *L.tacita* female.

### 

**Figures 1–10.**
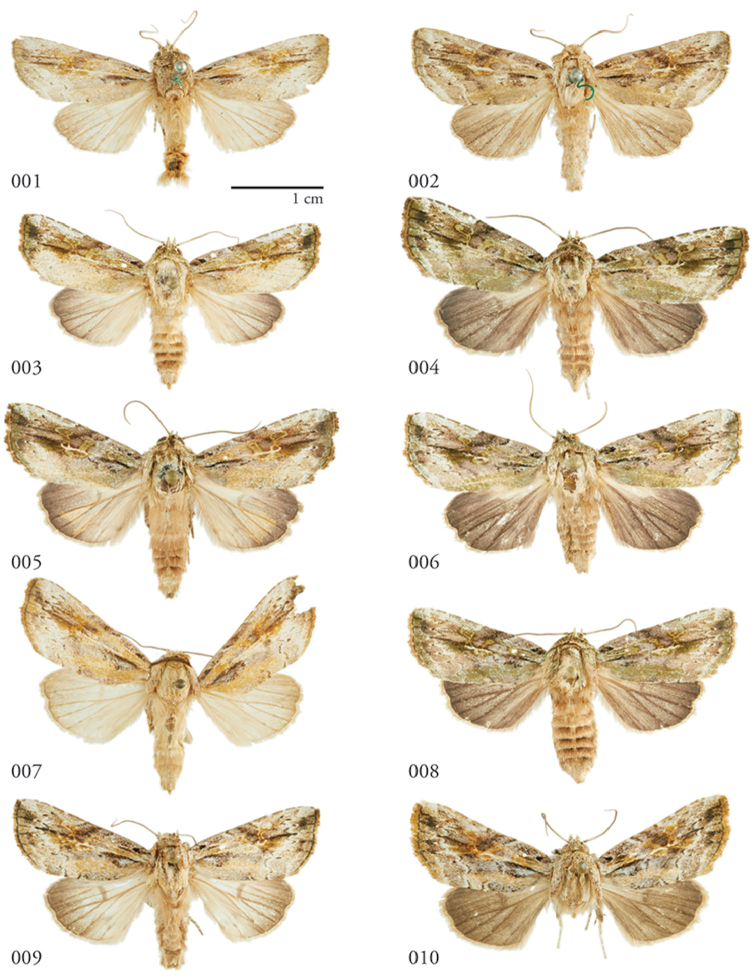
*Lophomyratacita*, dorsal habitus **1** ♂ Turrialba, Costa Rica, USNMENT01370323 **2** Holotype ♀, Costa Rica, USNMENT00973292, USNM Dissection 148201 **3** ♂ Área de Conservación Guanacaste (ACG), Costa Rica, 11-SRNP-70989, USNMENT01370310 **4** ♀ ACG, 10-SRNP-71914, USNMENT01370306 **5** ♂ ACG, 10-SRNP-73128, USNMENT01370305 **6** ♀ ACG, 10-SRNP-72638, USNMENT01370313, USNM Dissection 148145 **7** ♂ Venezuela, USNMENT01437226, USNM Dissection 148083 **8** ♀ ACG, 10-SRNP-70590, USNMENT01370320, USNM Dissection 148189 **9** ♂ Venezuela, USNMENT01370311 **10** ♀ Venezuala, USNMENT01370316, USNM Dissection148084.

**Figures 11–20.**
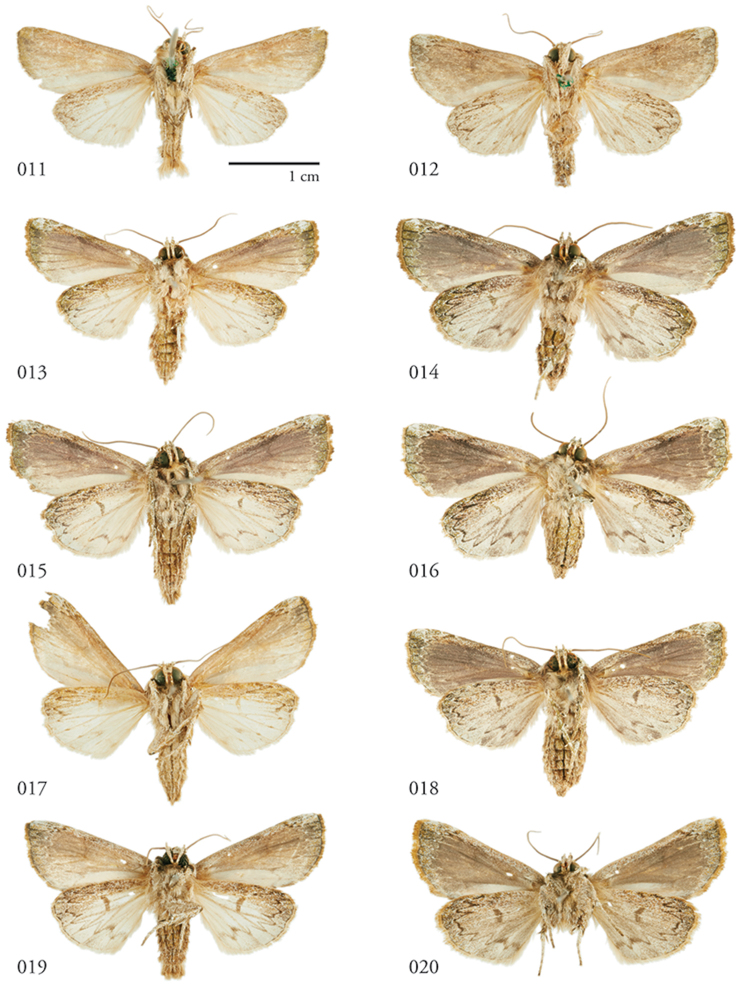
*Lophomyratacita*, ventral habitus. **11** ♂ Turrialba, Costa Rica, USNMENT01370323 **12** Holotype ♀, Costa Rica, USNMENT00973292, USNM Dissection 148201 **13** ♂ Área de Conservación Guanacaste (ACG), Costa Rica, 11-SRNP-70989, USNMENT01370310 **14** ♀ ACG, 10-SRNP-71914, USNMENT01370306 **15** ♂ ACG, 10-SRNP-73128, USNMENT01370305 **16** ♀ ACG, 10-SRNP-72638, USNMENT01370313, USNM Dissection 148145 **17** ♂, Venezuela, USNMENT01437226, USNM Dissection148083**18** ♀ ACG, 10-SRNP-70590, USNMENT01370320, USNM Dissection 148189 **19** ♂ Venezuela, USNMENT01370311 **20** ♀, Venezuela USNMENT01370316, USNM Dissection 148084.

**Figures 21–30.**
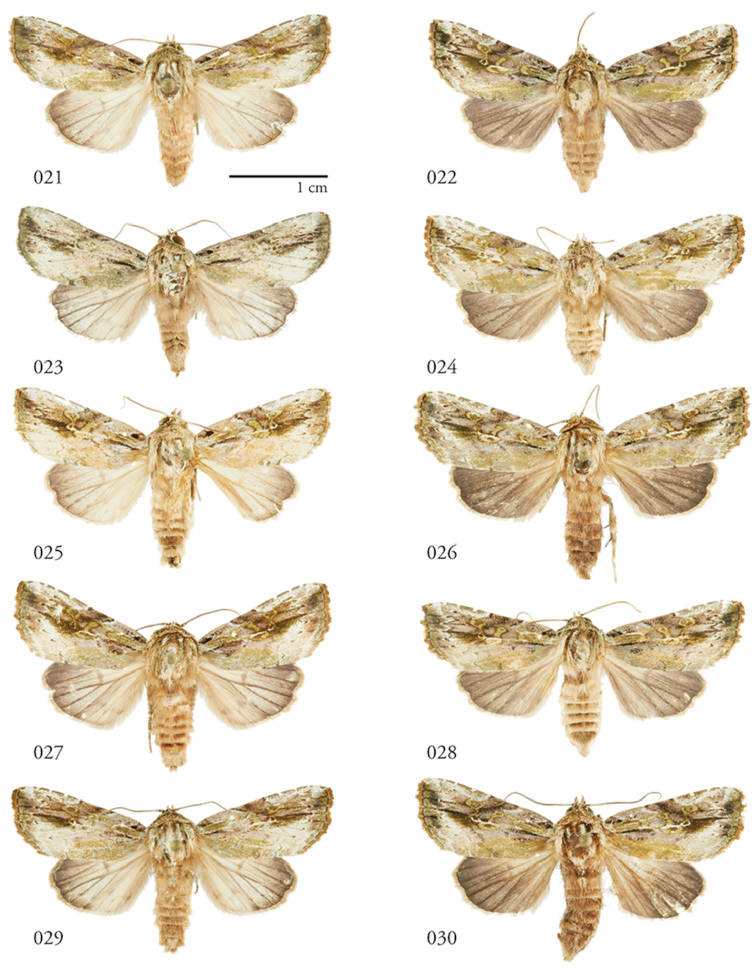
*Lophomyratacita*, dorsal habitus**. 21** ♂ Área de Conservación Guanacaste (ACG), Costa Rica, 10-SRNP-73286, USNMENT01370301 **22** ♀ ACG, 12-SRNP-1080, USNMENT01370309 **23** ♂ ACG, 13-SRNP-71895, USNMENT01370324 **24** ♀ ACG, 10-SRNP-73225, USNMENT01370322 **25** ♂ ACG, 10-SRNP-70707, USNMENT01370287 **26** ♀ ACG, 10-SRNP-70072, USNMENT01370280 **27** ♂ ACG, 10-SRNP-70496, USNMENT01370308 **28** ♀ ACG, 11-SRNP-70494, USNMENT01370312 **29** ♂ ACG, 10-SRNP-73310, USNMENT01437265 **30** ♀ ACG, 10-SRNP-70706, USNMENT01370302.

**Figures 31–40.**
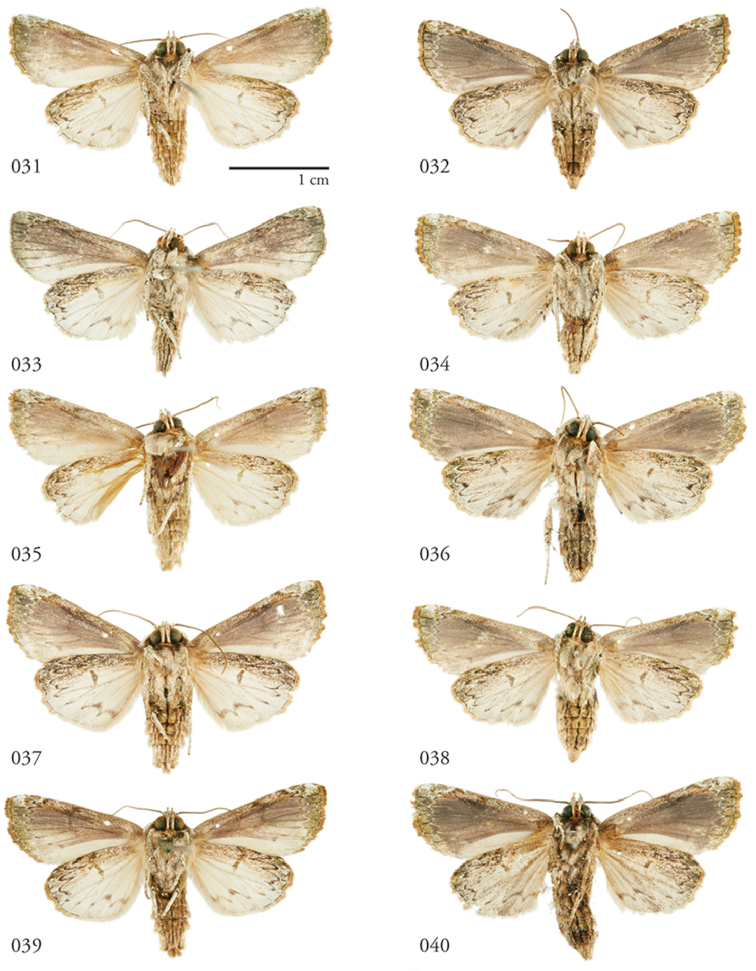
*Lophomyratacita*, ventral habitus **31** ♂ Área de Conservación Guanacaste (ACG), Costa Rica, 10-SRNP-73286, USNMENT01370301 **32** ♀ ACG, 12-SRNP-1080, USNMENT01370309 **33** ♂ ACG, 13-SRNP-71895 USNMENT01370324 **34** ♀ ACG, 10-SRNP-73225 USNMENT01370322 **35** ♂ ACG,10-SRNP-70707 USNMENT01370287 **36** ♀ ACG, 10-SRNP-70072 USNMENT01370280 **37** ♂ ACG, 10-SRNP-70496 USNMENT01370308 **38** ♀ ACG, 11-SRNP-70494 USNMENT01370312 **39** ♂ ACG, 10-SRNP-73310, USNMENT01437265 **40** ♀ ACG, 10-SRNP-0706, USNMENT01370302.

**Figures 41–48.**
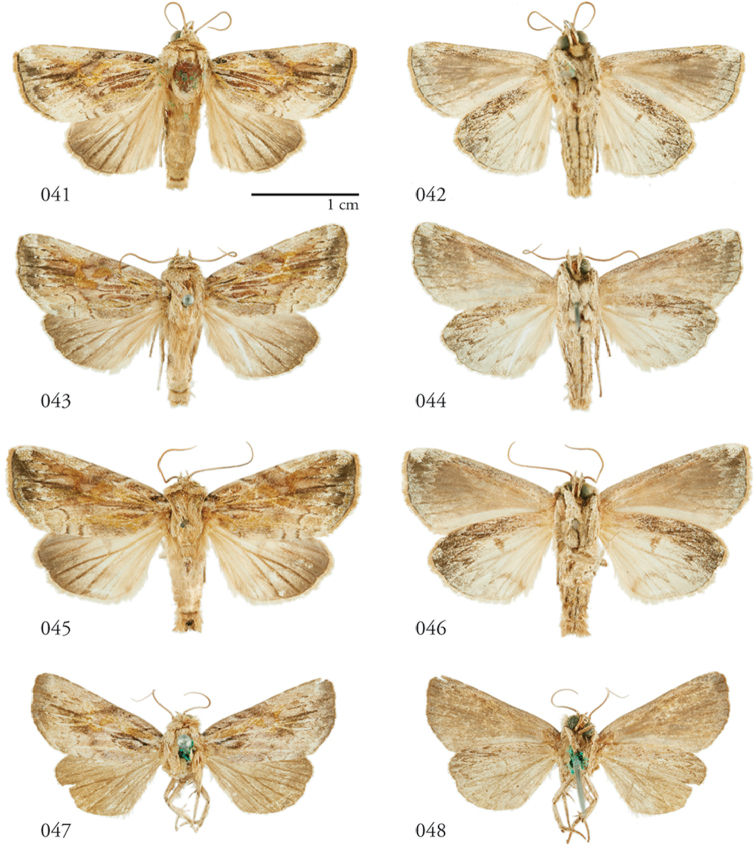
*Lophomyrasantista*, habitus. Dorsal (left), Ventral (right). **41, 42** Holotype ♂, Brazil, NHMUK01606195 **43, 44** ♂, Brazil, NHMUK01606196 **45, 46** ♂, Brazil, NHMUK01606198 **47, 48** ♀, French Guiana, USNM Dissection 148082, USNMENT01370318.

**Figures 49–56.**
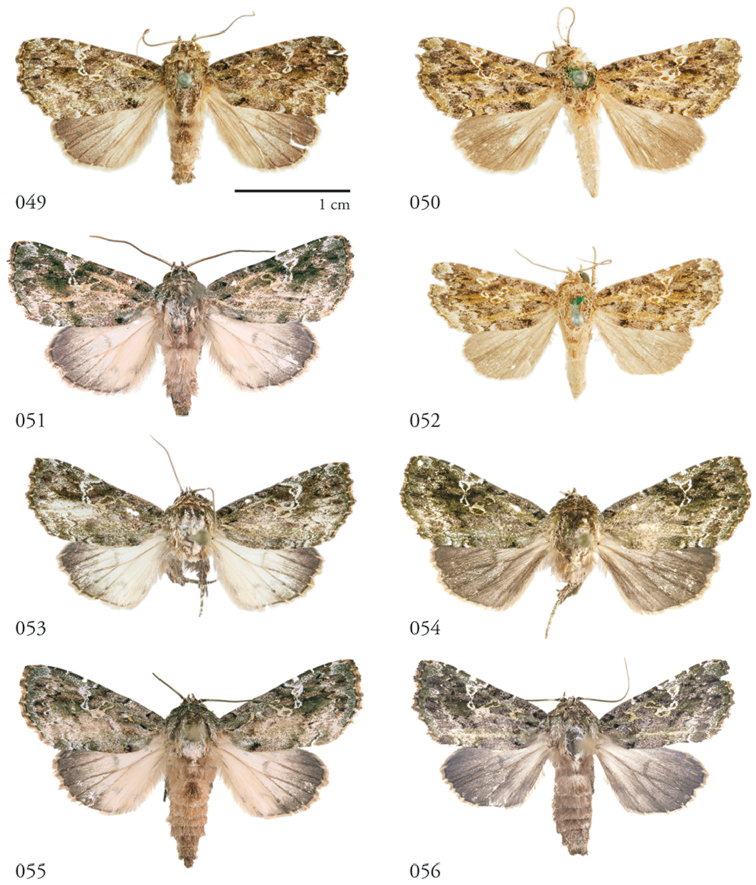
*Lophomyracommixta*, dorsal habitus. **49** Holotype ♂, French Guiana, USNMENT01370304, USNM Dissection 148186 **50** ♀ French Guiana, USNMENT01370319, USNM Dissection 148086 **51** ♂ Área de Conservación Guanacaste (ACG), Costa Rica, 11-SRNP-70741, USNMENT01370307 **52** ♀ French Guiana, USNMENT01370317 **53** ♂ ACG, 10-SRNP-70810, USNMENT01370328, USNM Dissection 148050 **54** ♀, ACG, 10-SRNP-70436,USNMENT01370329, USNM Dissection 148051 **55** ♂ ACG, 11-SRNP-69041, USNMENT01370325, USNM Dissection 148098 **56** ♀ ACG, 12-SRNP-70644, USNMENT01370326, USNM Dissection 148106.

**Figures 57–64.**
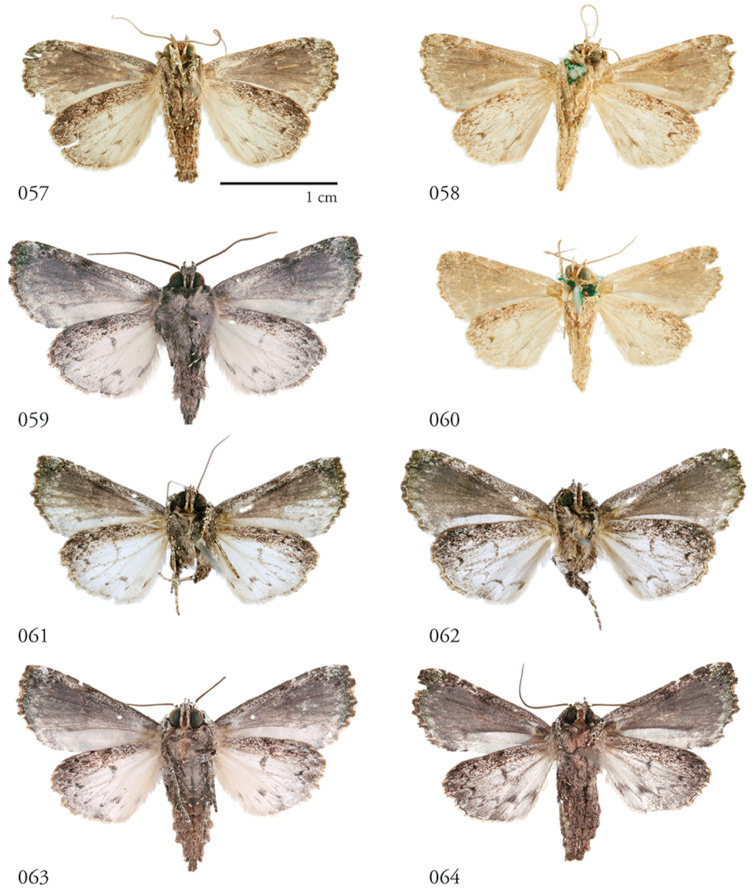
*Lophomyracommixta*, ventral habitus. **57** Holotype ♂, French Guiana, USNMENT01370304, USNM Dissection 148186 **58** ♀ French Guiana, USNMENT01370319, USNM Dissection 148086 **59** ♂ Área de Conservación Guanacaste (ACG), Costa Rica, 11-SRNP-70741, USNMENT01370307 **60** ♀ French Guiana, USNMENT01370317 **61** ♂ ACG, 10-SRNP-70810, USNMENT01370328, USNM Dissection 148050 **62** ♀ ACG, 10-SRNP-70436,USNMENT01370329, USNM Dissection 148051 **63** ♂ ACG, 11-SRNP-69041, USNMENT01370325, USNM Dissection 148098 **64** ♀ ACG, 12-SRNP-70644, USNMENT01370326, USNM Dissection 148106.

**Figures 65–72.**
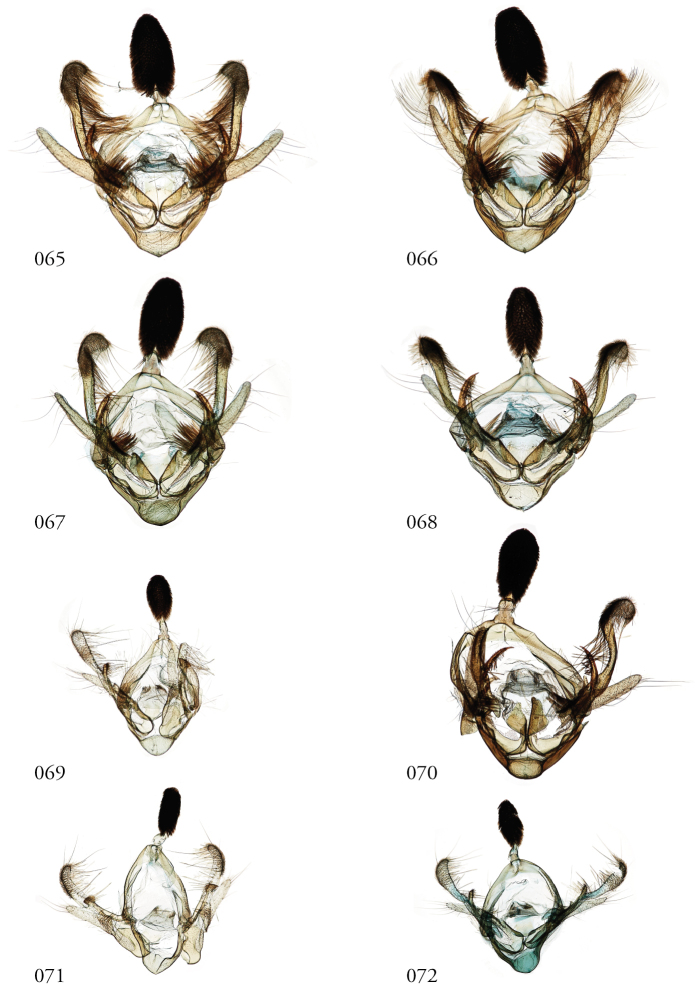
*Lophomyra* male genitalia, valves. **65***L.tacita*, Turrialba, Costa Rica, USNMENT01438834, USNM Dissection 148081, **66***L.tacita*, Turrialba, Costa Rica,USNM Dissection 148143, USNMENT01370321 **67***L.tacita*, Área de Conservación Guanacaste (ACG), Costa Rica, 10-SRNP-73310, USNMENT01437265 USNM Dissection 148099 *tacita***68***L.tacita*, Venezuela, USNMENT01437226, USNM Dissection 148083 **69***L.commixta*, Holotype, French Guiana, USNMENT01370304, USNM Dissection 148186 **70***L.santista*, Holotype, Brazil, NHMUK01606195 **71***L.commixta*, ACG, 11-SRNP-69041, USNMENT01370325, USNM Dissection 148098 **72***L.commixta*, ACG, 10-SRNP-70488, USNMENT01438848, USNM Dissection 148180.

**Figures 73–80.**
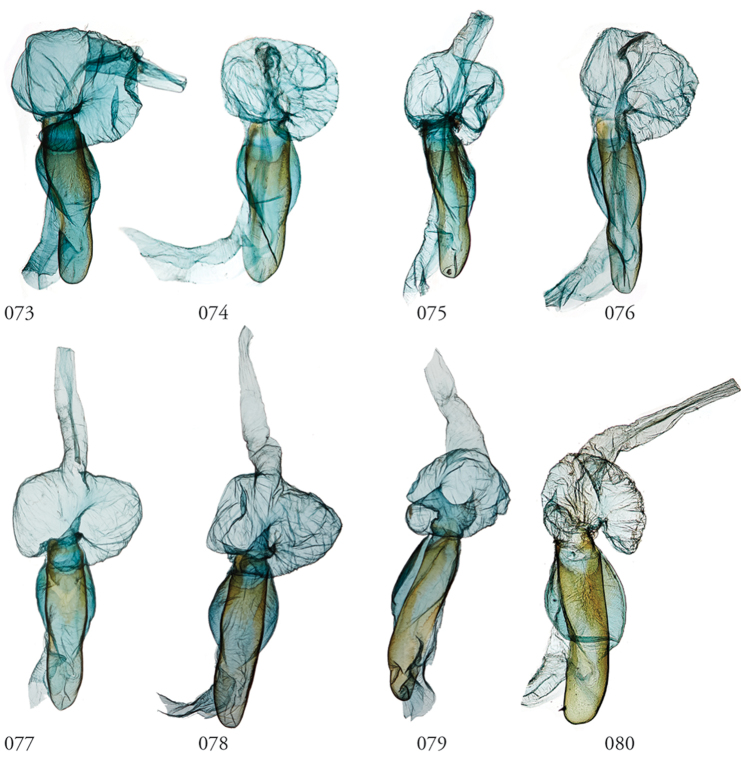
*Lophomyra* male genitalia, phalli. **73***L.tacita*, Turrialba, Costa Rica, USNM Dissection 148143, USNMENT01370321 **74***L.tacita*, Turrialba, Costa Rica, USNM Dissection 148081, USNMENT01438834 **75***L.tacita*, Área de Conservación Guanacaste (ACG), Costa Rica, 10-SRNP-73310, USNMENT01437265, USNM Dissection 148099 **76***L.tacita*, Venezuela, USNMENT01437226, USNM Dissection 148083 **77***L.tacita*, ACG, USNMENT01438858, USNM Dissection 148290 **78***L.tacita*, ACG, USNMENT01437222, USNM Dissection 148300 **79***L.santista*, French Guiana, USNMENT01438868, USNM Dissection 148301 **80***L.santista*, Holotype, Brazil, NHMUK01606195.

**Figures 81–84.**
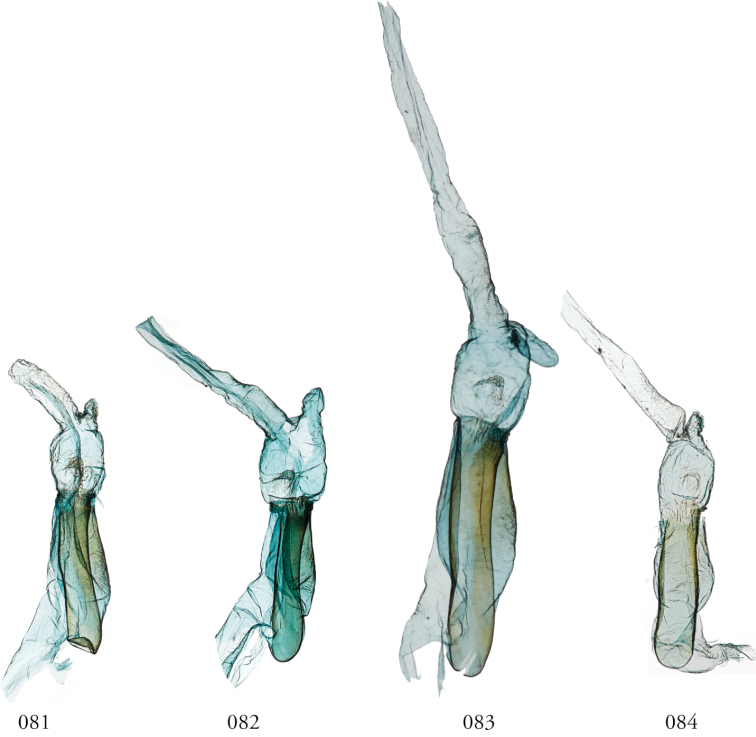
*Lophomyracommixta*, male genitalia, phalli. **81** Área de Conservación Guanacaste (ACG), Costa Rica, 11-SRNP-69041, USNMENT01370325, USNM Dissection 148098 **82** ACG, 10-SRNP-70488, USNMENT01438848, USNM Dissection 148180 **83***L.commixta*, USNMENT01437196, USNM Dissection 148299 **84***L.commixta*, Holotype, French Guiana, USNMENT01370304, USNM Dissection 148186.

**Figures 85–90.**
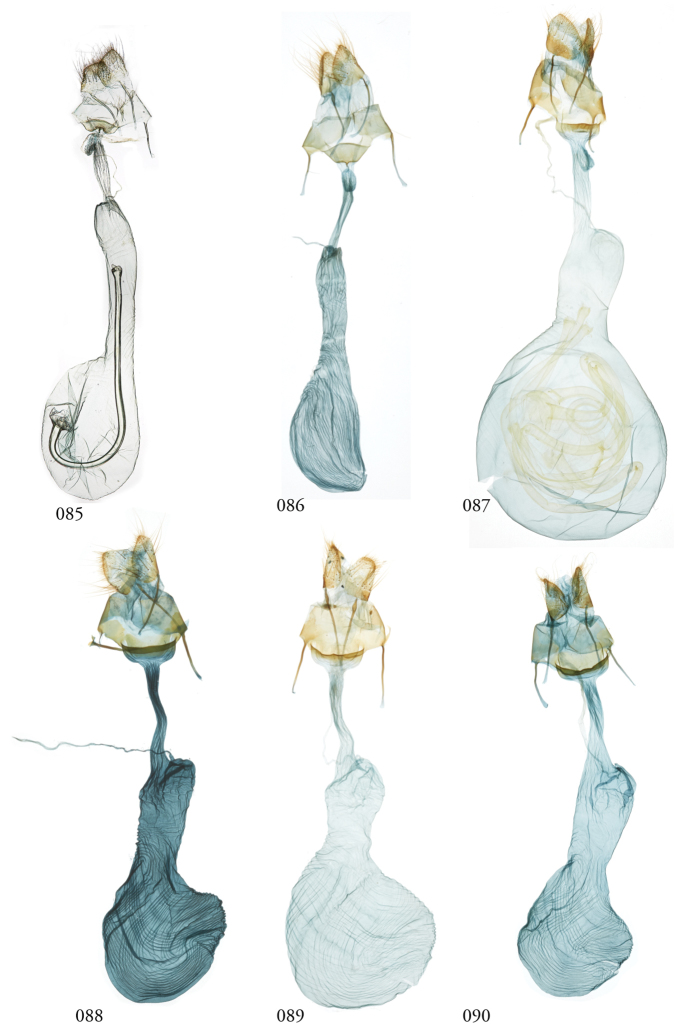
*Lophomyra* female genitalia. **85***L.commixta*, French Guiana, USNMENT01370304, USNM Dissection 148086 **86***L.commixta*, Área de Conservación Guanacaste (ACG), Costa Rica, 10-SRNP-70436,USNMENT01370329, USNM Dissection 148051 **87***L.santista*, French Guiana, USNM Dissection 148082, USNMENT01370318 **88***L.tacita*, ACG, 13-SRNP-71894, USNMENT01438803, USNM Dissection 148105 **89***L.tacita*, Venezuela, USNMENT01370316, USNM Dissection 148084 **90***L.tacita*, ACG, 10-SRNP-70460, USNMENT01437275, USNM Dissection 148053.

**Figures 91, 92.**
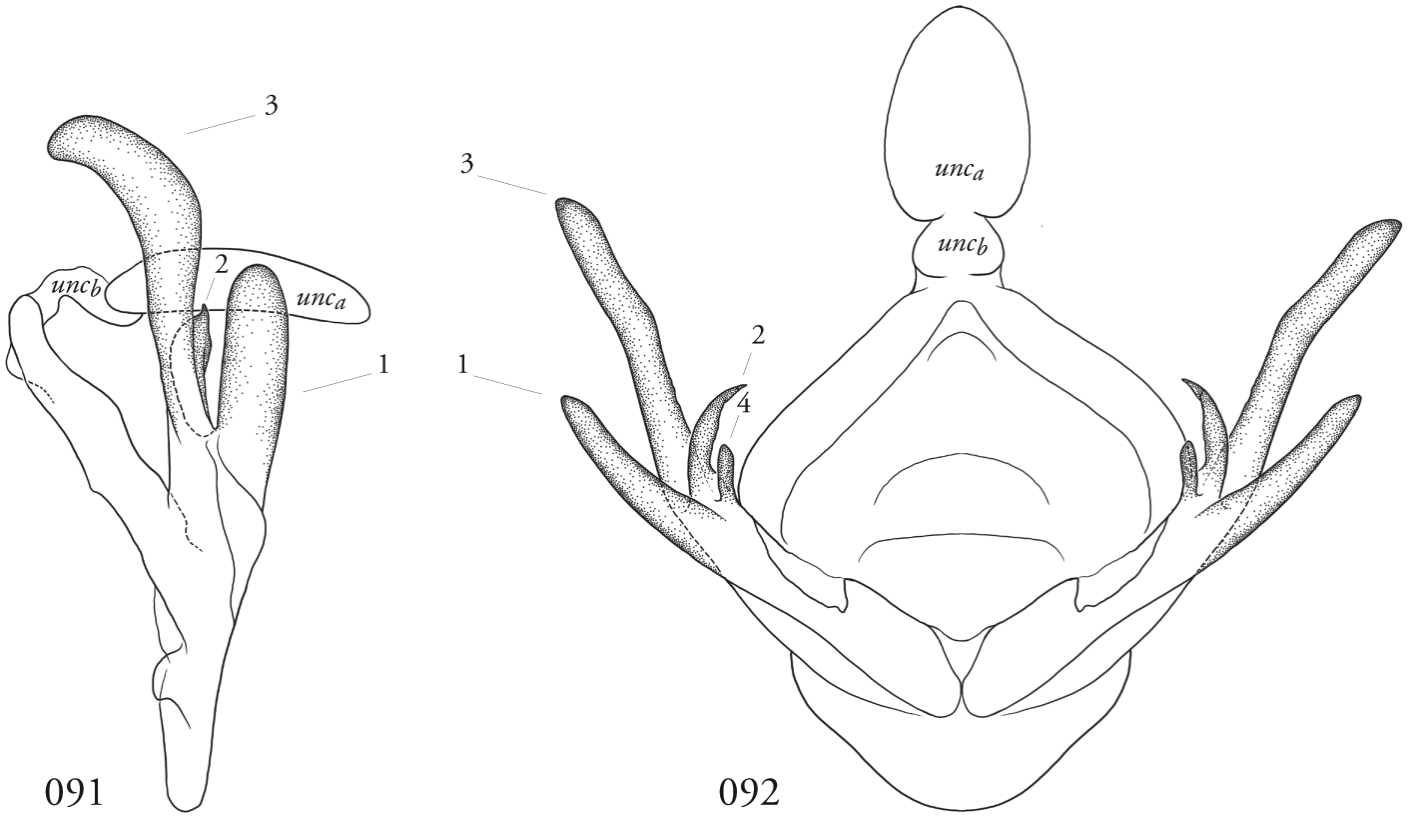
*Lomphomyratacita* male terminalia. Área de Conservación Guanacaste (ACG), Costa Rica, 10-SRNP-70435, USNMENT01437276, USNM Dissection 148202. **91** Lateral **92** Caudal. Numbers refer to structures as enumerated in text: 1 = sacculus; 2 = clasper; 3 = cucullus; 4 = ampulla-like process.

**Figures 93–99.**
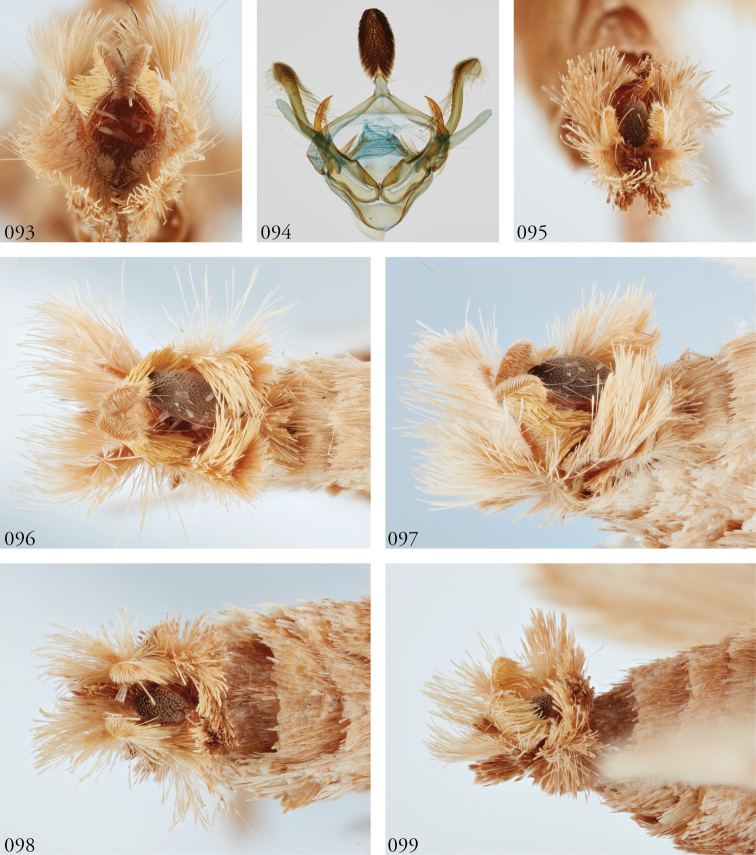
*Lophomyra* male terminalia *L.tacita* and *L.commixta***93***L.tacita*, Turrialba, Costa Rica, USNMENT01370323 **94***L.tacita*, Venezuela, USNMENT01437226, USNM Dissection 148083 VZ **95***L.commixta*, French Guiana, USNMENT01370327 **96***L.tacita*, Turrialba, Costa Rica, USNMENT01370323 **97***L.tacita*, Turrialba, Costa Rica, USNMENT01370323 **98***L.commixta*, French Guiana USNMENT01370327 **99***L.commixta*, French Guiana, USNMENT01370327.

**Figures 100–107.**
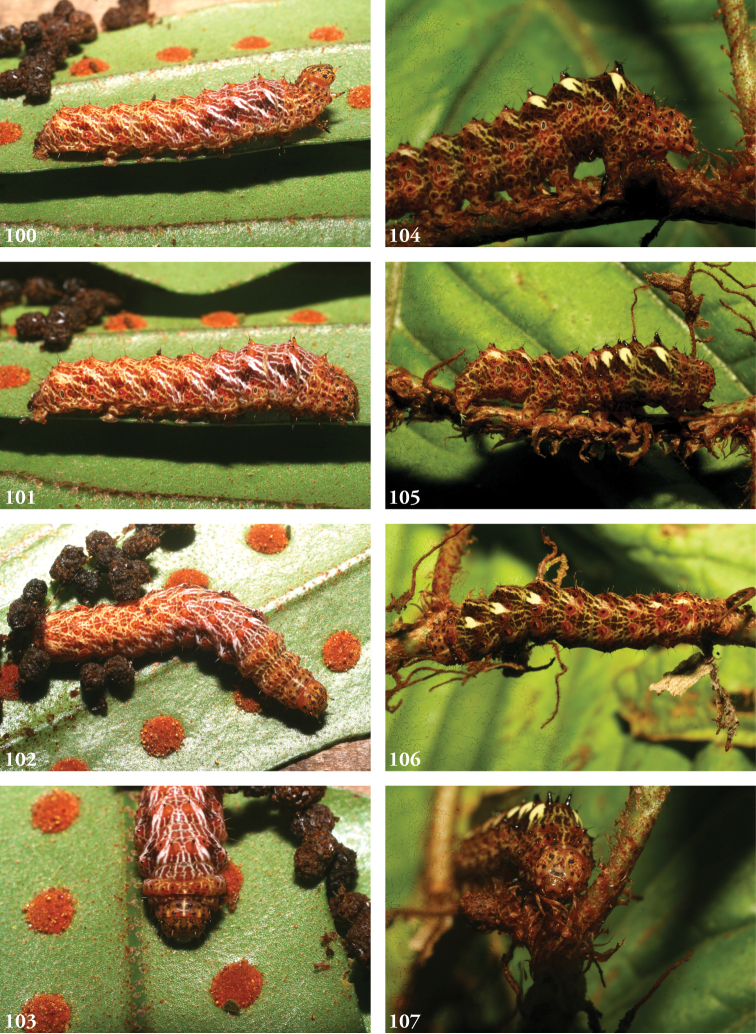
Larvae of *Lophomyracommixta*, Área de Conservación Guanacaste (ACG), Costa Rica. **100–103.** 11-SRNP-69041, USNMENT01370325, USNM Dissection 148098 (male), cf. Figs 55, 63, 71, 78**100** DHJ483519 **101** DHJ483520 **102** DHJ483513 **103** DHJ483516 **104–107.** 10-SRNP-70572, USNMENT01437261 (female) **104** DHJ469067 **105** DHJ469064 **106** DHJ469063 **107** DHJ469069.

**Figures 108–115.**
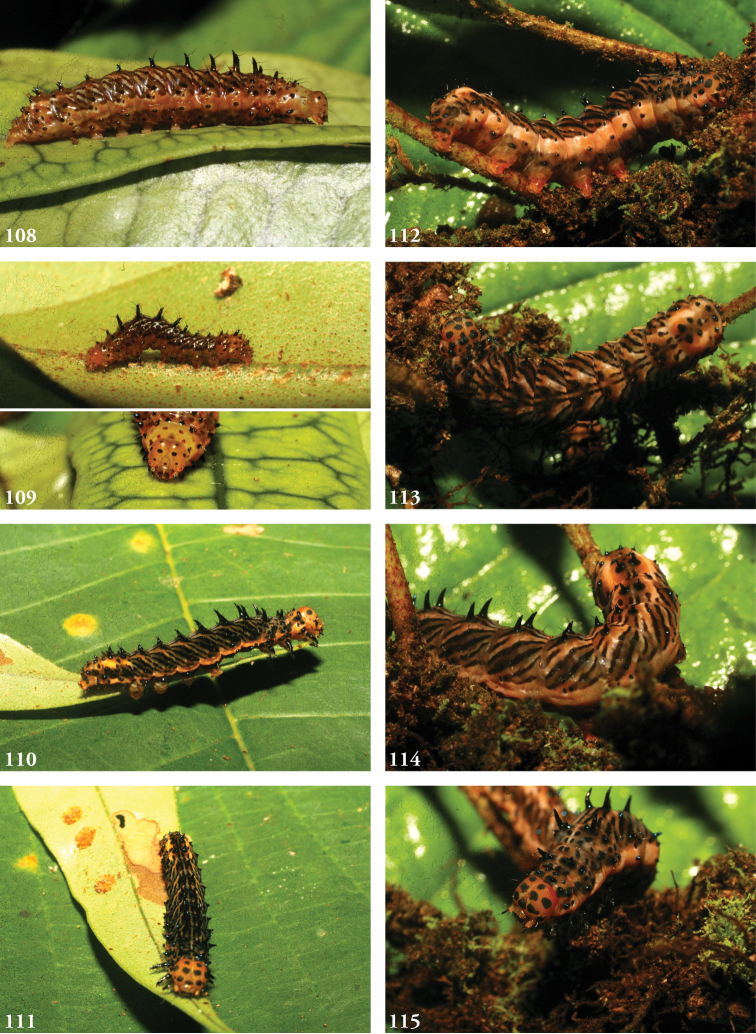
Larvae of *Lophomyratacita*, Área de Conservación Guanacaste (ACG), Costa Rica. **108** 10-SRNP-70396-DHJ469022, USNMENT01437191 (female) **109** 10-SRNP-70181-DHJ467611, USNMENT01437251 (top, male), 10-SRNP-70396-DHJ469019 (bottom, female) **110** 09-SRNP-73662-DHJ466954, USNMENT01437270 (female) **111** 09-SRNP-73662-DHJ466960, USNMENT01437270 (female) **112–115.** 10-SRNP-70001 (unreared) **112** DHJ467541 **113** DHJ467542 **114** DHJ467536 **115** DHJ467535.

**Figure 116:**
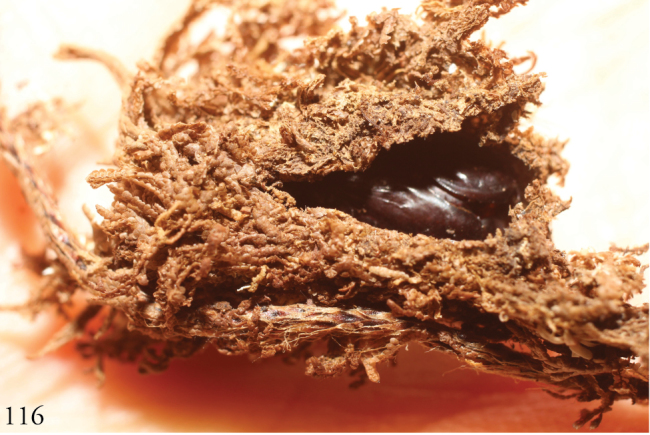
Cocoon of *Lophmyratacita* 10-SRNP-70471-DHJ498320.

**Figures 117–119.**
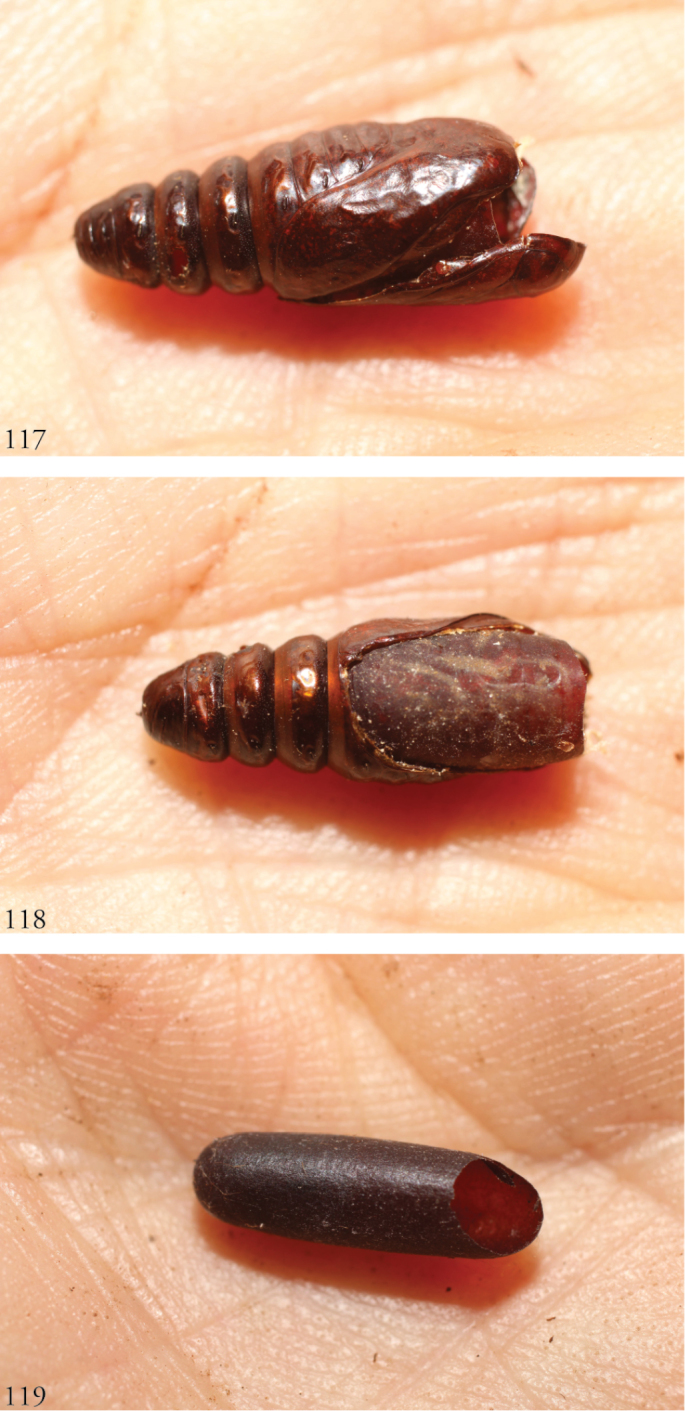
Parasitized pupae of *Lophomyratacita* and larva of *Lophomyracommixta*, Área de Conservación Guanacaste (ACG), Costa Rica. **117, 118***L.tacita*, 10-SRNP-70472 parasitized by undescribed species of *Atactosturmia* (DHJPAR0038699) (Tachinidae). **117** DHJ498322 **118** DHJ498324 **119***L.commixta*, 10-SRNP-71801-DHJ498385 *Diradops* (undescribed Banchinae) ichneumonid wasp cocoon from prepupal larva.

## Discussion

Although no sister-group relationship between *Lophomyra* and *Leucosigma* is indicated by analyses of COI barcode data, there are as yet insufficient data to determine whether their shared associations with ferns are independently derived or a function of common ancestry. Beyond their provisional assignment to the Noctuinae, we refrain from naming any higher-level taxonomic assignments. As is certainly the case for *Leucosigma*, there remain a number of cryptic species of *Lophomyra* yet to be described.

Among the more striking features of *Lophomyra* is the complex of what appear to be male courtship tufts and the unusual configuration of setae covering the uncus (which may serve as a pheromone-bearing structure as well) that diagnoses the genus. What appear to be shingled, dark-gray scales are revealed under high magnification to be palmate clusters of setae.

The known host plants of *Lophomyra* represent the phylogenetically narrowest diet breadth of any genus of fern-feeding noctuids thus far documented from ACG. All recorded hosts of *Lophomyra* are polypodiaceous ferns, which are among the more widespread hosts of known Neotropical pteridivorous noctuoid genera, most of which include species that have been recorded from Polypodiaceae, Dryopteridaceae, or both at ACG. These include the noctuid genera *Argyrosticta*, *Callopistria*, *Leucosigma*, and *Phuphena* and the erebid genera *Dusponera*, *Mamerthes*, *Nicetas*, *Rejectaria*, *Salia*, *Scopifera* and *Tarista*. With the exception of *Leucosigma*, each of these includes species that have been recorded from more than two fern families, and a majority from more than five.

## Supplementary Material

XML Treatment for Lophomyra

XML Treatment for Lophomyra commixta

XML Treatment for Lophomyra tacita

XML Treatment for Lophomyra santista
